# P013 Progressive Pregnancy-and Lactation-associated Osteoporosis (PLO) with Multiple Compression Fractures During Prolonged Breastfeeding

**DOI:** 10.1093/rap/rkag113.043

**Published:** 2026-11-03

**Authors:** Jessica Chan, Hlaing Myat Chit Su, Sian Griffith

**Affiliations:** East Surrey Hospital, Redhill, United Kingdom; East Surrey Hospital, Redhill, United Kingdom; East Surrey Hospital, Redhill, United Kingdom

## Abstract

**Introduction:**

Pregnancy-and Lactation-associated Osteoporosis (PLO) is a rare cause of fragility fractures in young women, most commonly presenting in the postpartum period with severe back pain and vertebral compression fractures. Diagnosis is often delayed due to overlap with common mechanical musculoskeletal pain following pregnancy and labour.

We report a case of progressive PLO in a young woman with multiple cumulative risk factors: prolonged lactation, use of therapeutic low-molecular-weight heparin (LMWH), paternal vertebral fracture, and vegan diet, complicated by prolonged breastfeeding, resulting in disease progression.

**Case description:**

A 33-year-old woman presented with severe, progressive back pain six weeks after delivery of her second child in February 2025. She had experienced a similar episode of back pain following the birth of her first child (May 2020), which was not investigated. Following persistence and worsening of symptoms, magnetic resonance imaging (MRI) four months postpartum demonstrated eight vertebral compression fractures involving T10 to L5. Dual-energy X-ray absorptiometry (DEXA) confirmed low lumbar spine bone mineral density (BMD) (Z-score –2.1), with preserved hip (Z-score –0.7) and femoral neck (Z-score 1.9). Serum biochemistry was unremarkable.

Her background included two provoked pulmonary emboli (PE), requiring lifelong coagulation. Her first PE followed immobility after foot surgery (2015), and due to strong family history of venous thromboembolism (father had three PEs, paternal uncle had one), warfarin was initiated. Due to non-compliance, she sustained a second PE (2017). She continued warfarin until her first pregnancy (2020), where she switched and remained on therapeutic enoxaparin through till 2026 because she continued breastfeeding her first child until 2024, and continued following the birth of her second child (2025). Additional to prolonged breastfeeding and long-term LMWH, risk factors for osteoporosis included a vegan diet, and a family history of osteoporosis (paternal osteoporosis aged sixty-six, maternal osteopenia). She had no other significant risk factors for osteoporosis. At the time of presentation, her medications included bisoprolol, pregabalin, inhalers, and venlafaxine.

After the discovery the initial fractures, she was commenced on calcium and vitamin D supplementation and advised stopping breastfeeding. She continued to breastfeed and unfortunately, repeat MRI seven months later (January 2026) demonstrated progression of existing fractures, with a new T8 compression fracture. She eventually stopped breastfeeding in June 2026 with the help of a charity and started on teriparatide. She is now anticoagulated with rivaroxaban.

**Discussion:**

PLO is a rare and under-recognised cause of vertebral fractures in young women. Diagnosis is often delayed as symptoms are often attributed to mechanical back pain, a common presentation in the early postnatal period affecting forty percent of women.

The pathophysiology is multifactorial. Pregnancy and lactation are associated with increased skeletal calcium mobilisation, reduced oestrogen levels, and increased bone turnover. In this case, continuous lactation of six years is likely to have been a significant contributory factor. PLO affects the axial skeleton, with relative preservation of hip and femoral neck bone density, as vertebral bone is more sensitive to hormonal changes. Therapeutic LMWH exposure is also a recognised potential contributor to reduced BMD, as well as strong family history, and vegan diet. In this case, multiple metabolic and iatrogenic factors likely acted synergistically rather than a single primary aetiology to increase fracture risk.

Standard management of PLO includes the cessation of breastfeeding alongside calcium and vitamin D supplementation. However, weaning is often complex and influenced by maternal preference, familial circumstances, and psychological factors. The World Health Organisation (WHO) recommends continued breastfeeding for up to two years of age or beyond, with some organisations encouraging even longer durations, such as up to seven years. Hormonal changes during the weaning of breastfeeding, including reduction in prolactin and oxytocin and fluctuation of oestrogen, may affect maternal mental health. The cessation of breastfeeding is linked to several biochemical and psychological factors, and patients with pre-existing mental health disorders may find this transition especially challenging.

This case illustrates the importance of early consideration of PLO in postpartum women with persistent or severe back pain, the need for prompt imaging, and recognition of cumulative risk factors. In this case prolonged breastfeeding was identified as a significant contributing factor.

**Key learning points:**

• Pregnancy-and lactation-associated osteoporosis (PLO) is an uncommon but important cause of vertebral fragility fractures in young women.

• PLO should be considered in postpartum women presenting with persistent, severe, or progressive back pain. This is especially relevant when symptoms are disproportionate to typical mechanical pain or fail to improve with conservative management.

• Spinal imaging should be performed promptly when clinical features are concerning, as vertebral fragility fractures may be missed. MRI is the preferred modality for identifying acute and subacute vertebral compression fractures.

• PLO is typically multifactorial, with physiological bone loss during pregnancy and lactation. This case was compounded by additional risk factors such as prolonged breastfeeding, medication-related effects, possibly low dietary calcium intake, and potential underlying genetic predisposition.

• Management of PLO should address reversible risk factors, such as cessation of breastfeeding. However, it is important to recognise that decisions regarding weaning are influenced by biological, psychological, and social factors.

